# Exploring the Potential of Microalgae as Feed Ingredients for Sustainable Aquaculture: A Review of Nutritional and Environmental Benefits

**DOI:** 10.1155/anu/5217142

**Published:** 2026-01-27

**Authors:** Mohamed Hachimi Alaoui, Aziz Elmoujtahid, Meriem Bamaarouf, Hicham El Arroussi

**Affiliations:** ^1^ Algal Biotechnology Laboratory, Moroccan Foundation for Advanced Sciences Innovation and Research (MASCIR), Mohammed VI Polytechnic University, Benguerir, Morocco, um6p.ma; ^2^ EMDD_CERNE2D, Higher School of Technology Sale, Mohammed V University in RABAT, 227 Avenue Prince Heritier, Sale, Morocco, um5a.ac.ma; ^3^ Environmental, Ecological and Agro-industrial Engineering Laboratory, Faculty of Sciences and Technics, Sultan Moulay Slimane University, Campus M’Ghila, P.O. Box 523,, 23 000, Beni Mellal, Morocco, universitesms.com; ^4^ National Agency for Water and Forests, Deroua Fish Farming Station, Beni Mellal, Morocco

**Keywords:** aquaculture, aquafeed, circular economy, microalgae, sustainability

## Abstract

The increase and rapid population growth and higher demand for fish are driving the aquaculture industry to rapid expansion. One of the main challenges in aquaculture is to ensure sustainable feeds for fish in different aquaculture systems. Historically, aquafeed ingredients were fishmeal and fish oil, but the increase in price and the decrease in availability have resulted in the utilization of some plant‐based aquafeed. One of the most utilized plant‐based aquafeed and alternative protein sources is soybean meal (SBM). However, the use of aquafeed high in plant protein affects the growth performance, and the production of SBMs requires arable land and freshwater that could be used for crops for human consumption. The substitute aquafeed must possess valuable content, including protein with essential amino acids and lipids with omega 3 fatty acids, and must be palatable and digestible, and it should have low levels of insoluble carbohydrates, fiber, and heavy metals, as these factors can impact fish growth and health. Focusing on microalgae as sustainable alternative has gained interest because microalgae naturally exist in aquatic food chains, with appropriate biochemical composition that could be suitable for lipid substitute in feed as well as fish oil, rich in proteins, carbohydrates, pigments, and other antioxidants. Microalgae, with this balanced composition and high biomass productivity, are considered as a potential aquafeed that can replace conventional ingredients. In this review, we describe how microalgae inclusion in aquafeed or as alternative to conventional sources is conduct to improve sustainability and quality of aquafeeds.

## 1. Introduction

The projected surge in the worldwide population to 9.3 billion by 2050 is a major challenge for food insurance [1]. Aquaculture is a crucial contributor to global food security, as it produces food for human consumption and is the most efficient source of edible proteins [2, 3]. Based on the FAO’s publication of 2024, the global production of aquatic animals has reached 185 million tons in the year 2022; of this total production, 94 million tons was from aquaculture and 91 million tons was from capture fisheries. World fisheries and aquaculture production shows 115 million tons in marine areas and 70 million tons in inland waters [4]. Due to the increase and rapid population growth, especially in many developed countries, the consumption of fish has increased and became the healthy alternative to some animal proteins and are the main dietary source of the eicosapentaenoic acid (EPA) and docosahexaenoic acid (DHA) [5–7]. To deal with the growing human population and the rising demand for fish, it is imperative to enhance the efficiency and sustainability of aquaculture [8]. Of the total aquatic animal produced, 89% was used for human consumption, and the rest went to nonfood uses, mostly fishmeal and fish oil [4].

Aquaculture industries are facing many challenges, especially with the supply of feed ingredients, and require feeds that improve the health and quality of produced fish [9]. The composition of aquafeed depends on the nutritional requirements of farmed species [10], and traditionally farmed fish have been nourished with diets containing fishmeal as protein‐rich ingredient and fish oil as a source of omega 3 long‐chain polyunsaturated fatty acids (PUFAs; EPA and DHA) [11]; due to its beneficial characteristics, balanced biochemical composition, concentrated vital components, and excellent effects on fish growth and well‐being, it continues to be the most desired feed ingredient [12, 13] because it is very palatable and provides essential amino acids and essential fatty acids as well as highly digestible energy [14]. Fishmeal and fish oil are produced from whole fish, fish trimmings, or fish by‐products to ensure stable production, reducing waste and improving resource valorization [4]. The availability of feed such as FM and FO are progressively constricted due to limited resources; consequently, alternative sources of feed ingredients are being explored, notably increasing interest towards replacing FM and FO with plant‐based alternative [15, 16]. Soybean is a major source of protein for human and source of vegetable oil [17]; among all plant protein sources, soybean meals (SBMs) are a prominent plant‐based protein source that is widely used in aquaculture feeding, and its incorporation into fish diets is proposed to decrease feed costs [18].

Nevertheless, the use of aquafeed containing high levels of plant protein can impact the growth performance of aquatic animals [19, 20]. In addition, soybean production is also linked with environmental impacts, including extensive deforestation and greenhouse gas emission [21, 22].

As stated earlier, the higher demand, the limited supply of FM and FO, and the high prices to produce these ingredients have led to the search for new sustainable ingredients [23]. Sustainable aquafeed means using nutritionally balanced fish feed that minimize reliance on wild fish and support involvement of alternative ingredients [24]. Therefore, to meet the need for feed with health benefits, alternatives must guarantee important criteria such as nutritional profile, palatability, and digestibility and must be both economically and environmentally sustainable [25, 26].

The increasing demand of aquafeed has led to find other sustainable alternatives, and the inclusion of microalgae shows potential for substituting FM and FO in aquafeeds [27]. Multiple research studies have demonstrated the successful integration of microalgae biomass as a feed supplement or substitute for FM in different aquaculture species. These investigations consistently provide positive results in terms of promoting growth and improving overall quality [28, 29]. The inclusion of microalgae in aquaculture has gained considerable attention, offering numerous advantages; with their photosynthetic capacity requiring light and source of carbon (CO_2_) for growth in photoautotrophic conditions, they can grow rapidly and produce biomass without requiring arable land and competing with agricultural activities [30, 31]. Moreover, some microalgae can grow heterotrophically or mixotrophically depending on different sources of carbons [32, 33], for heterotrophic microalgae use organic compounds for both energy and carbon, which produces biomass in dark conditions [34], whereas mixotrophic microalgae utilize both light energy and external organic carbon for generating high biomass yield [35]. Microalgae present high diversity species ranging from 200,000 to 800,000 species [36]; several ones could be found in marines or freshwater environment [37], showing considerable adaptability in harsh environmental conditions [38, 39], and could be cultivated in wastewater for bioremediation with the capacity of removing nutrients, metals, and biomass production [40, 41].

Highlighting the microalgae biomass composition, several microalgae strains are rich in various high value compounds such as proteins which can reach 70% in *Spirulina platensis* [42], *Spirulina* proteins for human have gained popularity as a food supplement [43], carbohydrates in *Chlorella vulgaris* can reach 35% [44] and lipids with high value unsaturated fatty acids EPA and DHA [45, 46], many microalgae species are capable to accumulate lipid, under certain stressful conditions can reach considerable amount, and in certain oleaginous microalgae lipid content can reach 90% under nitrogen deprivation [47, 48]. In addition to the abovementioned metabolites, microalgae biosynthesize minerals, vitamins, and pigments with considerable biological effect [49]. Pigments such as phycocyanin from *Arthrospira platensis*, carotenoids from *Dunaliella salina*, and astaxanthin from *Haematococcus pluvialis* [50, 51] have various properties such as antioxidant activity, anti‐inflammatory, and immunostimulant effect [52–54].

Microalgae as a source of feed in aquaculture have gained importance, and the balanced nutrients of various compounds of microalgae biomass can meet nutritional requirements of aquatic animals. This research aims to assess the feasibility of utilizing microalgae biomass as sustainable ingredients in aquaculture feed as well as the benefits associated with its use. This review aims to describe the potential of microalgae and their rich nutritional profile and low environmental footprint, as promising solution to mitigate the negative impacts of conventional fish feed sources, such as FM and FO. This knowledge is crucial to produce healthy fish with exceptional quality standards and optimized welfare circumstances.

## 2. Sources of Conventional Feeds and Their Limitations

With the tremendous growth of the aquaculture sector, the dependance on aquafeed supplies is also increasing [55, 56]. In 2023, global aquafeed production was estimated at 69.42 million tons, and it is projected to reach 92.73 million tons by 2030 [57]. In previous research publications, the commercial feed usage by fed‐aquaculture species between 2000 and 2018 was estimated by 13.83 and 52.74 million tons, respectively [58]; fishmeal and fish oil are used as ones of the mains ingredients in fed aquaculture [59]; however, several aquafeed industry, for example, Norwegian salmon, has promoted to reduce reliance to these limited resources and minimize their inclusion [58, 60].

The feeds must fulfill the specific dietary requirements of aquatic animals, including carbohydrates, proteins, lipids, minerals, and vitamins. Aquafeeds commonly source from a diverse range of organisms, including animals, plants, and microorganisms to meet these criteria. This guarantees the provision of essential ingredients for the health and survival of aquatic species. The sustainability of fish feed relies heavily on feed efficiency and the selection of appropriate feed materials [13, 23]. Aquafeed heavily relies on FM and FO, derived from wild‐caught forage fish; these components serve as the primary source of protein and lipid in aquaculture feeds [29, 61]. FM is a rich protein ingredient; it is produced from complete fish or unwanted fish by‐products [62] that are notably distinguished as the most superior protein origins in aquafeeds and is characterized by a well‐proportioned amino acid profile that is highly digestible, while FO contains advantageous phospholipids and long‐chain PUFAs [8, 63, 64]. The use of fishing by‐products significantly contributes to environmental protection, and by‐products can account for up to 70% of fish and shellfish following industrial processing, with significant emphasis placed on transforming these into marketable products [65, 66]. Fish waste has emerged as a significant issue with challenges that must be addressed; a lot of fish parts such as heads, frames, viscera, and skin which are not consumed as human food may be repurposed for aquafeed production and can be transformed into fishmeal or fish oil; these by‐products are abundant in proteins, lipids, minerals, and vitamins [63]. For example, tilapia has a modest fillet yield of 33%, since tilapia residues do not reach human consumption standards and can be used to produce fishmeal [67].

Balanced dietary intake has become a crucial limiting element to improve the quality and efficiency of production [68]. In 2020, the production of aquatic animals was 178 million tons, and by 2030, it is forecast to reach 202 million tons [8, 69], and the demand for aquafeed is expected to increase due to its increasing application in the cultivation of various aquatic species, such as tilapia, carp, catfish, and salmon [70].

The sustainability of aquaculture systems is contingent upon various economic, social, resource‐related, and environmental aspects. Economic and social factors, such as economic development, food security, and employment, may promote aquaculture development, while resource constraints, including land, freshwater, and fishmeal, may hinder it. Additionally, environmental factors, such as ecological and water footprints, along with policy considerations, are also critical determinants [71], alongside rising energy costs and escalating demand, which have led to a global surge in fishmeal and fish oil prices. Consequently, extensive research has concentrated on substituting these aquafeeds with more economical and potentially less environmentally detrimental ingredients, such as plant by‐products [72].

During the past two decades, an important number of studies have been conducted to explore the potential of plant‐based ingredients as a substitute for the progressive replacement of traditional marine‐derived ingredients (FM and FO) in the formulation of aquafeeds [73]. Utilizing alternative plant products will decrease feed expenses and reliance on fishmeal, enhancing economic advantages [74]. Plant protein sources generally include soybean, canola, maize, potato protein, rice, lupin meal, guar meal, almond meal, and other ingredients that have been employed as aquaculture feed [75, 76].

Plant protein‐based alternatives have demonstrated efficacy in promoting optimal growth in fish; nevertheless, they also have certain drawbacks. Plant‐based protein may exhibit low digestibility; lack key critical amino acids such as lysine, methionine, threonine, and tryptophan; and can lead to considerable alterations in the nutritional content of the final fish products [77–79]. Plants also have antinutritional factors and a high proportion of cellulose [79, 80]. SBM is the foremost protein source, characterized by a balanced amino acid profile, affordability, and a reliable supply, rendering it an optimal substitute for fishmeal [81]. Soybeans contain 41% protein on a dry matter basis [82]. Nevertheless, SBMs have low palatability, inadequate essential amino acid content, and contain various antinutritional factors, such as phytates, tannins, trypsin inhibitors, and oligosaccharides [83]. The fish’s quality may decline due to the difficulty of digesting plant‐based protein and the absence of essential amino acids [15].

Due to the increasing growth of aquaculture, higher demand, decreased availability, and rising prices of fishmeal and fish oil, sustainable aquaculture feed alternatives are needed [23]. The aquaculture sector is growing due to human consumption, so it is a must to find sustainable feed ingredients to replace fishmeal, fish oil, and SBM [13]. Alternative fish feed should be of high quality and nutritional value, with omega 3 fatty acids, high protein content, sufficient amino acids, and digestible [23, 84].

Several novel dietary ingredients are currently with a potential for incorporation in aquafeeds; with a combination of protein, fat, carbohydrates, and other beneficial substances, microalgae biomass is a promising feed source that might satisfy this demand [84].

## 3. Microalgae as Sustainable and Alternative Fish Feed

Microalgae and cyanobacteria are the most abundant and diversified photosynthetic microorganisms with the capacity to synthesize nutritional and bioactive compounds [85–87]. Microalgae are photosynthetic microorganisms that basically utilize nutrients, carbon dioxide, and sunlight to generate biomass [88, 89], they are characterized by their rapid growth rate compared to terrestrial plants without requiring arable land, and they can grow in extreme conditions and harsh environment [90–93]. With these characteristics, microalgae are also explored to extract high value compounds such as pigments and long‐chain fatty acids which are useful in different domains [85, 94]. Microalgae are organisms recognized for their biotechnological and ecological importance [95, 96] and have become sustainable strategies due to their ability to synthetize a wide variety of biological active compounds [97–99].

In aquaculture, microalgae are considered as natural food for zooplankton in their natural ecosystems and extensively used to feed fish larvae, crustacean’s larvae, and mollusks [100, 101], and they are known as the primary producer for many aquatic animals [102]. Some microalgae are rich in protein, lipids, and essential nutrients such as pigments that could impact positively on the aquatic animals and presents advantages to be utilized as source of aquafeed [103–106]. In 2019, global microalgal biomass production was reported at 56,465 tons in 10 countries, which the vast majority was produced in China, this production encompasses *Spirulina* by 56,208 tons, *Haematococcus pluvialis* 242 tons, *Chlorella vulgaris* 4.77 tons, *Tetraselmis* spp. 1.45 tons, and *Dunaliella salina* 0.22 tons [107]. Different strains of microalgae in their biochemical content have slightly different compositions which can be a point of selection according to needs [108, 109]. Microalgae selection depends on the application and is specific for certain substances, *Dunaliella salina* is known for its β‐carotene content which can reach 14% of dry weight [110] with several effects as an antioxidant and have an effect on pigmentation of aquatic animals [111], and equally, astaxanthin from *Haematococcus pluvialis* is largely used for enhancing pigmentation in salmon [112].

Microalgae are rich in proteins, lipids, and bioactive compounds such as PUFAs, astaxanthin, and phycocyanin, which are essential to the growth and health of aquatic animals (Table 1) [103–105], play an important role in aquaculture systems, are considered as natural sources of nutrition, and could be used as a growth and immune stimulant given their content of antioxidants [109, 126]. Marine fish species have little capacity to synthetize these fatty acids such as omega 3 LC‐PUFAs, EPA, and DHA [127, 128] that are relying on dietary intake basically from FM and FO [129]; microalgae as a promising source fatty acids have the capacity to satisfy the needs and provide essential nutrients for the growth of aquatic animals, playing a key role in their appearance and physiological development [100, 130].

**Table 1:** Some dietary ingredients produced by microalgae and potential application for aquafeed.

Microalgae species	Bioactive compounds	References
*Arthrospira platensis* and *Nannochloropsis gaditana*	Proteins	[113, 114]
*Chlorella vulgaris*	Carbohydrates	[115]
*Dunaliella salina*	Exopolysaccharides	[116]
*Isochrysis galbana* and *Phaeodactylum* sp.	Lipids	[117, 118]
*Schizochytrium* sp. and *Crypthecodinium cohnii*	Essential PUFAs	[119]
*Haematococcus pluvialis*	Asthaxanthin	[120]
*Spirulina platensis*	Phycocyanin and phycoerythrin	[121]
*Dunaliella salina*	β‐Carotene	[122]
*Muriellopsis* sp.	Lutein	[123]
*Chlorella* sp., *Arthrospira* sp., *Isochrysis galbana*, *Pavlova* sp., and *Tetraselmis* sp.	Vitamin A, B12, C, E, K	[124, 125]

**Figure 1:**
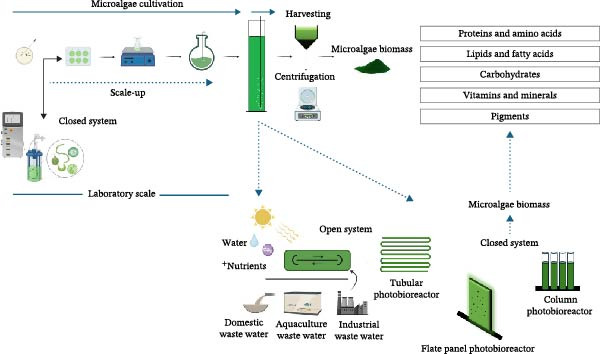
Schematic representation of different microalgae cultivation systems in laboratory and large scale for biomass production.

Some microalgae deserve more focus due to their potential and uses as aquafeed [106]. Microalgae such as *Arthrospira platensis*, *Chlorella* sp., *Pavlova* sp., *Dunaliella* sp., *Haematococcus* sp., and *Nannochloropsis* sp. are mostly utilized in aquafeeds due to their nutritional content and potential uses as new ingredients to formulated aquafeed [15, 131]. Microalgal biomass proteins varies from 60% to 71% in *Arthrospira platensis*, *Chlorella vulgaris* contains 51%–58% protein [132, 133], other microalgae such as *Isochrysis galbana* also belong to the group of promising microalgae in aquafeed due to its high lipid contents with LC‐PUFAs mainly EPA and DHA [91, 127], and supplementing aquafeed with abovementioned fatty acids improves higher omega 3 fatty acids in fishes [134]. Microalgae are a natural source of highly interesting biologically active compounds (Figure 1) [135]. Biotechnologically microalgae species can produce in harsh environments such as temperature, light intensity, and salinity, different metabolites with antioxidant activity, pigments, and lipids which are used in several industrial products [136].

In aquaculture industries, microalgae have been investigated for use in aquafeed formulation and are an excellent alternative to fishmeal and fish oil as a source of protein and lipid [13, 137]. Due to its high and valuable content of proteins, carbohydrates, and lipids, in the early stages of development, marine microalgae provided essential nutrition and is currently used as feed for culture and feeding larvae and juvenile shellfish [138, 139], and in addition to these primary metabolites, microalgae synthesize a wide variety of secondary metabolites that generally include pigments and vitamins [49, 140].

Inclusion of microalgae in fish feed has the potential to meet demand for sustainable aquafeed; with their high value composition and benefits, they have attracted attention for replacing fishmeal and SBM, and microalgae could be involved in fish feed in different ways and different forms including extruded pellets and microalgal paste [141–143]. Several studies have been reported the effects of inclusion of microalgae biomass in aquatic animal’s diets on growth performance (Table 2).

**Table 2:** Examples of the effects of inclusion of microalgae on growth performance of aquatic animals.

Aquatic animals	Microalgae species	Inclusion level (%)	Replacing	Weight gain (%) (microalgae‐based diet)	Weight gain (%) (reference diet)	FCR (microalgae‐based diet)	FCR (reference diet)	SGR (microalgae‐based diet)	SGR (reference diet)	References
*Dicentrarchus labrax*	*Nannochloropsis* sp.	10	Fish oil and wheat	246	239	1.16	1.15	1.68	1.65	[144]
*Litopenaeus vannamei*	*Schizochytrium* sp.	7.5	Fish oil	637	562	2.07	2.16	2.36	2.24	[145]
*Litopenaeus vannamei*	*Aurantiochytrium* sp. meal	8	Fish oil	293	297	2.10	2.08	5.66	5.38	[146]
*Oreochromis niloticus*	*Chlorella* sp.	15	Fishmeal	38	22	1.24	1.78	0.51	0.32	[147]
*Oreochromis niloticus*	*Nannochloropsis gaditana*	30	Whole	212	212	0.90	0.96	2.76	2.71	[148]
*Oncorhynchus mykiss*	*Scenedesmus* sp.	5	Fishmeal and fish oil	104	108	1.14	1.15	1.8	1.9	[149]
*Salmo salar* L.	*Schizochytrium* sp.	5	Fish oil	426	326	0.90	0.90	1.5	1.3	[150]
*Salmo salar* L.	*Scenedesmus* sp.	10	Fishmeal	95	107	0.88	0.76	1.03	1.12	[151]
*Solea senegalensis*	*Tisochrysis lutea*	15	Fishmeal and fish oil	318	161	1.28	0.93	1.68	1.69	[152]
*Sparus aurata*	*Nannochloropsis gaditana*	5	Soybean	102	98	1.09	1.16	1.96	1.9	[153]
*Sparus aurata*	*Tetraselmis* sp.	10	Soybean	245	248	1.40	1.42	2.03	2.04	[154]

Abbreviations: FCR, feed conversion ratio; SGR, specific growth ratio.

## 4. Microalgae for the Substitution of Aquafeed

Fishmeal is considered as a crucial constituent in aquafeed due to its well‐balanced biochemical profile with a high protein and essential amino acids content, lipids, and omega 3 fatty acids, in addition to their palatability and digestibility [13]. Microalgae also possess several advantages, for example, *Spirulina*, *Chlorella*, and *Nannochloropsis*, containing protein up to 70% with a good balance of essential amino acids [155, 156]. For their high protein contents, some microalgae such as spirulina and chlorella inclusion in fish diet of certain aquatic animals show positive effects with amelioration of growth performance, which suggest the possibility to replace fishmeal [2]. For replacing FO in aquafeed, microalgae due to their lipids content can reach a total lipid content of 45% or even exceed 60% in some oleaginous species under environmental conditions and could be a promising candidate for replacing FO in aquafeed [5, 143, 157].

In addition to the abovementioned compounds of microalgae, they are enriched with a wide range of other high value compounds, including natural pigments, for example, *Dunaliella salina* synthetizes β‐carotene, and this pigment allows the application of *Dunalliella salina* in aquafeed to improve the immune response and the color degree of aquatic animals [158, 159]. *Haematococcus pluvialis* also synthetizes astaxanthin; when applied, it ameliorates the pigmentation [160]. Some microalgae can synthetize essential vitamins such as vitamins A, B (B1, B2, B6, and B12), C, D, and K [161–163], which are essential for growth and immunity [164, 165].

Microalgae biomass could be used as a supplement and mixed with fishmeal in fish diet preparation to ensure nutritional requirements of fish species and aquatic animals (Table 3) [174].

**Table 3:** Examples of some cases of utilization of microalgal biomass for fishmeal replacement in aquafeed and specific effect on aquatic animals.

Algae species	Inclusion level in diet	Aquatic animal	Specific effects	References
*Chlorella* meal	Fishmeals replaced by: 75% 100%	Crucian carp (*Carassius auratus*)	Amelioration of growth and feed utilization *Chlorella* meal can be used as a protein source alternative	[166]
*Chlorella vulgaris*	Fishmeal replaced by: 5% 15% 25%	African catfish (*Clarias gariepinus*)	Improvement of feed intake, weight gain, and protein efficiency ratio *Chlorella vulgaris* is a suitable source of protein in the diet of African catfish	[167]
*Spirulina platensis* meal	Fishmeals replaced by: 2.5% 5%, 7.5 % 10%	Rainbow trout (*Oncorhynchus mykiss*)	High weight gain by inclusion of 7.5% *Spirulina platensis* meal Inclusion of 10% results in a high redness and yellowness of fish fillet	[168]
*Spirulina platensis* meal	Fishmeal replaced by: 2.5% 5% 10% 20%	Three‐spot gourami (*Trichopodus trichopterus*)	Enhancement of growth parameters of fish by inclusion of 10% Fish growth and fecundity were impacted negatively by inclusion of 20% Inclusion at 8.1% to 9.6% in fish diet is optimum for fishmeal replacement	[169]
*Nannochloropsis* sp. and *Isochrysis* sp.	Fishmeals replaced by 15%	Juvenile Atlantic cod (*Gadus morhua*)	Improvement of fish’s growth parameters	[170]
*Arthrospira fusiformis*	Supplementation with 1% of *Arthrospira fusiformis*	*Oreochromis niloticus* (Nile tilapia)	Higher specific growth rates, feed conversion ratio and average weight gain	[171]
*Spirulina platensis* and *Chlorella vulgaris*	Fishmeals were replaced with diets containing 50% and 75% *Spirulina platensis* and 50% and 75% *Chlorella vulgaris*	African catfish (*Clarias gariepinus*)	Diets substituted with both microalgae species ameliorate the growth performance	[172]
*Schizochytrium* sp.	Inclusion of 8% of dried biomass	*Oreochromis niloticus* (Nile tilapia)	Improved growth parameters and increase in DHA content in the fillet lipids	[173]

## 5. Bioavailability and Digestibility of Microalgal Nutrients

In several fish species, microalgae are increasingly studied as functional feed additives [175], with high values for fish in sustainable aquaculture; microalgae possess an interesting biochemical composition which are essential in fish diets [176]; microalgae composition varies by species, generally includes, for example, proteins (up to 70% in *Spirulina*), carbohydrates (up to 55% in *Chlorella vulgaris*), and lipids (up to 60% in *Nannochloropsis*) [177–179]; this composition could vary depending on conditions of cultivation and can be changed in favor of valuable product [180].

With all these valuable biochemical compositions, in aquaculture feeds, one of the main challenges is to offer feed with bioavailable and digestible nutrients, and these parameters can significantly affect fish growth and health. Microalgae nutrient bioavailability can vary based on species, and the rigid cell walls of some microalgae species, such as *Chlorella*, mainly composed with cellulose, can limit nutrient absorption [143, 181]. Cyanobacterial biomass like *Spirulina* is easy to digest as its cell wall lacks polysaccharides and is made up of mucopeptide easily utilized by fish [134, 182]. Microalgae can ensure protein requirements of fish with improved digestibility compared to plant protein [183]. Processing techniques which aim to cell wall disruption can improve digestibility and nutrient bioavailability [143]. Several studies investigated correlation between nutrients accessibility and their digestibility; in this context, Teuling et al. [148] tested how mechanical and physical *Nannochloropsis gaditana* cell wall breaking can improve nutrient accessibility and their digestibility for *Oreochromis niloticus* juvenile; the results showed that the treated microalgae increase the apparent digestibility of protein from 62% to 78% and lipids from 50% to 82%; similarly, Sessegolo Ferzola et al. [184] and Van De Walle et al. [185] investigated the effect of processed *Chlorella vulgaris* and freeze dried and spray dried on digestibility and growth when used for tilapia feed; results showed that spray drying makes nutrients bioavailable to fish and improved protein and energy digestibility.

Microalgae are known for their antioxidants, antimicrobial, and anti‐inflammatory properties [109, 126]. Marine fish species have little capacity to synthetize these fatty acids such as omega 3 LC‐PUFAs, EPA, and DHA [127, 128] that are relying on dietary intake basically from FM can improve fish health and immunity [137]. The inclusion of microalgae in fish diets can enhance growth by improving feed conversion efficiency and nutrient utilization, especially when processed to enhance digestibility [175]. Different processing methods can enhance the nutritional value and digestibility of microalgal biomass. For example, preextrusion can improve dry matter digestibility without affecting protein digestibility [3].

**Figure 2:**
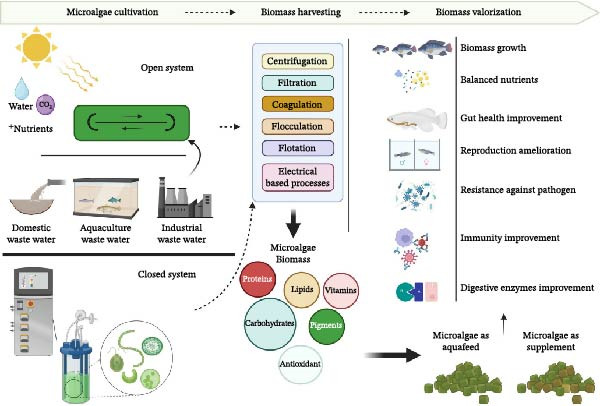
Microalgae biotechnology and benefits of administration of microalgae biomass in aquafeed.

Microalgal biotechnology has emerged as a focal point of research across multiple disciplines, owing to the diverse bioproducts derived from these microorganisms [135, 186]. In aquaculture, microalgae administration in fish diets can enhance growth and improve disease resistance, immune response, and different parameters for fish well‐being (Figure 2) [187].

## 6. Microalgae and Fish Health

Microalgae biocompounds act synergistically to fortify fish immunity through antioxidant defense, immune cell activation, and anti‐inflammatory modulation, offering sustainable alternatives to chemical treatments in aquaculture [188]. Microalgae‐derived bioactive compounds enhance fish immunity through multiple mechanisms due to their composition with antioxidants such as astaxanthin from *Haematococcus pluvialis*, a powerful antioxidant that enhances stress tolerance and immune function by reducing oxidative stress and improves antioxidant capacity, protecting immune cells from damage and enhancing resistance to pathogens [189], and phycocyanin from *Arthrospira platensis* enhances mucosal immunity and disease resistance in fish and shrimp [190]. From *Chlorella*, polyphenolic compounds which are well‐known for their antioxidant properties, including flavonoids and phenolic acids, can neutralize free radicals and have various health benefits [191]; polyphenols act as potent antioxidants, reducing oxidative stress in fish tissues and scavenging free radicals. This helps protect immune cells and maintain overall health [192]. These compounds reduce reliance on antibiotics by enhancing innate immunity [193].

**Figure 3:**
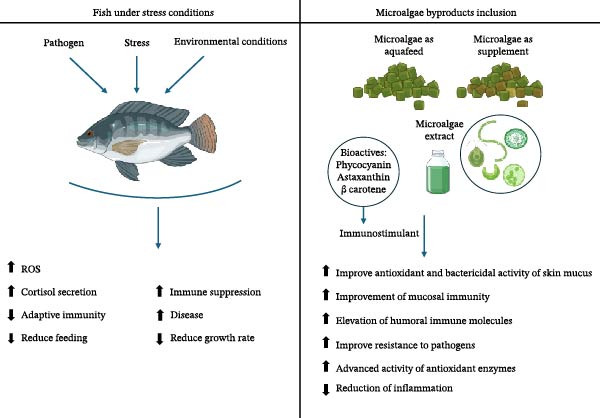
Inclusion of microalgae in fish diets and their roles in improving stress tolerance, inhibiting oxidative stress, and ameliorating immune responses.

Microalgae with their bioactive compounds are highly valuable in aquaculture in terms of their capacity to stimulate immune functions; the inclusion of *Phaeodactylum tricornutum* as a dietary supplement of the gilthead seabream (*Sparus aurata* L.) enhances immune activity such as phagocytic and hemolytic activities [194]; in addition, microalgae bioactive molecules reduce oxidative stress and improve disease resistance [195] (Figure 3).

The use of immunostimulants such as β‐glucan and its incorporation in diets or uses such as adjuvants has been suitable in aquaculture [196, 197], for example, a study evaluated the effects of chrysolaminarin rich extract derived from *P. tricornutum*, and the results show a high antioxidant activity and immunostimulant activity after in vivo experiment in Senegalese sole [198]. Another study has evaluated the effects of inclusion of microalgae such as *Schizochytrium* sp., *Spirulina platensis*, and *Dunaliella salina* on intestinal health and immunity; the results show a decrease in proinflammatory cytokines in intestine, indicating the reduction on inflammation [156].

Fish diseases are one of the challenges facing aquaculture industries [199], attributing to significant economic losses and negatively impacting humans by the consumption of contaminated fish [200, 201]. *Vibrio* spp. are abundant pathogens found in many aquaculture systems [200], and *Vibrio* species cause vibriosis in fish, leading to remarkable damages including loss of appetite, lethargy, skin ulcerations, hemorrhage, gill and fin lesions, and mortality [199, 202]. *Vibrio vulnificus* is one of vibrio pathogens [199], a study showed that inclusion of live *Isochriys galbana* in culture could serve as a biocontrol agent in aquaculture systems to reduce *Vibrio vulnificus* contamination, and this is due to bioactive molecules and secondary metabolites derived from microalgae with antibacterial effects, serving both bivalves and fish [203, 204]. In addition, fish could be exposed in their environment to other several pathogens affecting immune depressed fish, Okasha et al. [205] investigated the effect of inclusion of *Spirulina platensis* in Nile tilapia feed challenged against Edwardsiellosis, the results showed that the group given 10 g *Spirulina platensis* per kilogram of feed have improved growth and feed utilization and provided a relative protection level of 22.2% against the infection, and the normal status of fish was restored.

### 6.1. Effect of Aquafeed Based in Microalgae on Gut Microbiota

The gut microbiota with diverse communities is crucial for nutrition metabolism and physiological functions [206]. Healthy gut microbiota is extremely important in maintaining the well‐being of organisms [207, 208]. In aquaculture systems, the composition of fish’s gut microbiota is relative to many factors that influence variability and abundance of microbial communities, and one of the main factors is diet [209]. The study of Liu et al. [210] analyzed the gut microbial communities and compared in the same habitat between carnivorous and herbivorous fishes, they found that herbivorous fishes rich in clostridium, citrobacter as cellulose degrading bacteria, while in carnivorous fishes, cetobacterium and halomonas were dominant. Replacement of feed ingredients shows impact in intestinal microbiota of fish, and several studies investigated the effect of alternatives to fishmeal on gut composition, for example, in the study of Li et al. [211] shows the change of gut microbiota composition of Atlantic salmon (*Salmo salar* L.) feeding with an insect diet, with increase of bacillus in the gut.

**Figure 4:**
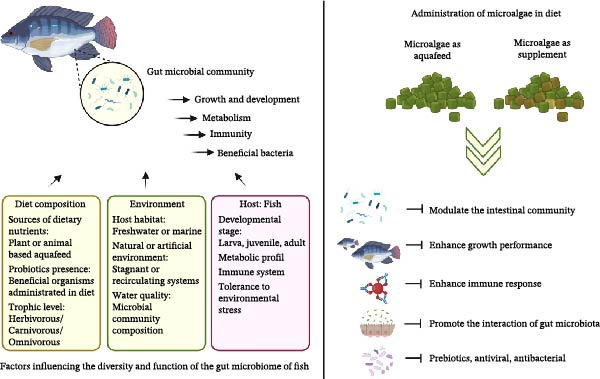
Factors influencing gut microbiota and microalgae administration in diet with promising modulation effects.

The uses of microalgae‐based aquafeed show promising effects on gut microbiota (Figure 4). Several studies show that inclusion of microalgae in aquafeed enhances gut health, supports beneficial microbial population, and promotes resistance to pathogens [212, 213]. For example, inclusion of 0.6% of *Haematococcus pluvialis* improved the composition of gut microflora of crayfish and promoted its growth and gut development [214]. Huang et al. [215] studied the impact of inclusion of 2% of *Chlorella vulgaris* on the diversity of gut microbiota. The results indicated that during the feeding times of 30 days, *Chlorella vulgaris* may promote the interaction of gut microbiota [215]. Ma et al. [212] studied the effect of inclusion with *Schizochytrium* sp., *Spirulina platensis*, *Chlorella sorokiniana*, *Chlorella zofingiensisi*, and *Dunaliella salina* could modulate the intestinal community and could enhance growth performance and immune response in zebrafish.

One of the main challenges facing intensive aquaculture industries is infectious disease and dependance to antibiotic [216], microalgae application is emerging as promising functional aquafeed with the potential to act as probiotics and prebiotics [217]. Probiotics are defined as live microorganisms that, when administered in adequate amounts, provide a health benefit on the host [218], and probiotics have the capacity of promoting immune system, improving nutrients digestibility, and promoting the development of healthy gut microbiota [219]. The use of microalgae as probiotics is limited because of the difficulty to maintain viability, primarily due to gastrointestinal acidic pH [220]. Prebiotics are known as any compounds that serve as substrates for the enhancements of the growth and functionality of gut microorganisms [221, 222]; microalgae contain several bioactive compounds that act as prebiotics stimulating the growth and development of beneficial gut microorganisms [223]; for example, microalgae nondigestible polysaccharides serve as prebiotic substrates for gut microorganisms and improve immune system [217].

## 7. Quality of Fish Products

Microalgae can significantly influence the quality of fish meat by affecting its flavor, texture, color, and fatty acid profile. Microalgae are rich in omega 3 fatty acids, particularly EPA and DHA, which are essential for human health. When included in fish feed, these microalgae enhance the omega 3 content in fish fillets, improving their nutritional value [224]. Microalgae like *Haematococcus pluvialis* contain astaxanthin, a carotenoid pigment that enhances the red or pink coloration of fish flesh, particularly in salmonids. This improves the visual appeal of the fish [225]. Microalgae enhance the nutritional profile of fish by increasing protein content and improving fatty acid composition, which can indirectly affect muscle texture. For example, a diet rich in omega 3 fatty acids from microalgae can improve the overall health and integrity of fish muscle, potentially leading to better texture [224]. Kousoulaki et al. [226] proved that the inclusion 5% *Schizochytrium* sp. improved lipid retention efficiency especially EPA and DHA, improving the gaping reduction in the fillet of Atlantic salmon (*Salmo salar* L.). Similarly, Sáez et al. [227] assess the effect of the inclusion at moderated level, up to 5%, of *Nannochloropsis gaditana* in the diets of gilthead seabream (*Sparus aurata*); the results indicate the enhancement of quality parameters, texture of fillets, and fatty acid profile [227].

## 8. Environmental Benefits of Microalgae in Aquaculture Compared to Conventional Ingredients

To guarantee the sustainability of aquaculture, evaluation of the environmental impact of the production of conventional feed is essential [228]; the production of fishmeal and fish oil is principally related to fishing [229]; and fishmeal production industries are associated with high energy consumption, high carbon dioxide emission, and ecosystems degradation [230–232]. The process of production of FM and FO from fishing to production factories is energy‐intensive and greenhouse gas generating [230]. Parker et al. [233] indicated that in 2011, fisheries consumed 40 billion liters of fuel and generated 179 million tons of CO_2_ [233, 234], and also the production of fishmeal and fish oil generates significant emissions of greenhouse gases, ~320 kg and 4430 kg of CO_2_ equivalent by producing one ton of fishmeal and one ton of fish oil, respectively [231]. Replacing fishmeal and fish oil by plant‐based ingredients, similarly, had significant impacts; soybean cultivation requires arable land; the depletion of freshwater resources and the expansion of soybean production lead to deforestation [22, 235]; the results of life cycle assessments (LCAs), collected and analyzed by Lucić et al. [236], estimate the emission of greenhouse gases with a global warming potential associated with soybean production on farms between 0.27 and 1.53 kg of CO_2_ equivalent per kg of soybeans produced [236]. In terms of climate change, Lehuger et al. [237] estimated for SBM production the global warming impact is 391 kg CO_2_ equivalent per ton of treated soybean. Microalgae offer several environmental benefits in aquaculture compared to conventional ingredients. Microalgae absorb carbon dioxide and release oxygen through photosynthesis, contributing to carbon sequestration [25], and can be cultivated without dependence on freshwater resources; it can be cultivated using wastewater, and consuming wastewater nutrients lead to reduced pollution and improved water quality [225]. Microalgae had high productivity and can grow in various culture systems that provide biomass with high‐value composition, making microalgae a sustainable source for aquafeed production [84, 238].

## 9. Perspectives and Challenges

Alternative ingredients in fish feed and any substitute should be high in nutrients, including proteins with amino acids, lipids with fatty acids, and carbohydrates, and with lower antinutritional elements, nonsoluble carbohydrates, fiber, and heavy metals that negatively affect fish growth and cause undesired waste [13]. Microalgae offer several nutritional advantages; they are a rich source of abovementioned ingredients [239]; because of microalgae composition, increasing attention regarded this resource as a potential ingredient and efficient alternatives to conventional aquafeed [165]. In addition to nutritional values, microalgae improve the health of aquatic species due to several metabolites with antioxidant capacity, immunostimulant activity, and the ability to promote physiological functions [190].

Environmentally, microalgae can sequester carbon dioxide, can assimilate nutrients from wastewater, and can be cultivated in extreme conditions [240]; in addition, some microalgae species combine the advantages to grow according to photoautotrophic, heterotrophic, or mixotrophic cultivations, allowing microalgae the capacity according to their metabolic specifications to generate biomass for multiple applications especially as aquafeed [241].

Despite the abovementioned advantages of microalgae and their potential application as aquafeed, many challenges are facing their inclusion. Firstly, the cost of production of microalgae biomass including cultivation, harvesting, and processing must be optimized [242]; the cost of microalgal production varies according to cultivations systems, open or closed systems; these two different systems had different productivity and different quality of produced biomass [243]; difference depends on nutrient supplies, also on the control level of microalgae culture in terms of avoiding contamination in large scale [244]. Concerning microalgae production cost, for example, the production cost of *Spirulina* and *Chlorella* exceeds 5 USD and 10 USD per kilograms, respectively [165], the search of cost competitive replacement of FM and FO has become indispensable, the study of Sarker et al. [3] investigated the substitution of FM and FO by protein‐rich defatted *Nannochloropsis oculata* and *Schizochytrium* sp. respectively to produce a fish‐free feed for Nile tilapia, the results shows competitive microalgae‐based feed, and the median feed cost of fish‐free feed was (0.68 USD/kg feed) compared to reference feed by (0.64 USD/kg feed).

In integrated biorefinery approach, microalgae with their capacity to grow in wastewater could be cultivated in aquaculture effluents containing nutrients required for growth; thus, the biomass produced could be used as aquafeed in terms of minimizing cultivation cost of microalgae [245]. Inclusion of microalgae in the process of treating streams from recirculating aquaculture system (RAS) has gained interest; microalgae could utilize nutrients from these effluents and produces biomass with high amount of proteins; the study of Vázquez‐Romero et al. [246] demonstrated that the incorporation of microalgae to treat *Solea senegalensis* streams promotes the treatment of the effluents by removing around 90% of nutrients including phosphorus and nitrogen; this is not only to obtain treated effluents but to produce protein‐rich biomass that could replace fishmeal by up to 21.4% in *Solea senegalensis* fattening feeds [246].

To benefit from the entire nutritional values of microalgae, nutrients must be bioavailable and digestible [143]; digestibility of microalgae biomass in aquatic animals is one of the main challenges; some microalgae have cell walls that affect the digestibility and make nutrients availability difficult [15]; to improve digestibility, an additional step, such as extrusion and enzyme supplementation, must be added in aquafeed production process [15, 247].

Microalgae present a promising source of sustainable aquafeed, they provide essentials nutrients with beneficial effects on growth performances and health of aquatic animals and could substitute various commercial ingredients; however, the promotion of this alternative must be more efficient by selecting appropriate microalgae with beneficial composition with more palatability and digestibility, optimizing cultivation and harvesting system to meet lower production cost, and integrating bio circular economy approach to enhance benefice and sustainability of aquaculture systems.

## 10. Conclusion

The application of microalgae as aquafeed ingredients has shown significant potential due to their tremendous advantages. Microalgae biomasses are known by high value components with high biological effects; in aquaculture systems, introduction of microalgae as aquafeed can improve growth parameters and health of produced aquatic animals and minimize dependance to conventional aquafeeds. However, microalgal biomass production requires controlled conditions to produce high‐quality biomass, and also their applications in aquafeed require nutrients accessibility and bioavailability to guarantee effectiveness of aquafeed. Despite their advantages, the high production cost, nutrient bioavailability, and digestibility are the main challenges in the application of microalgae. To ensure a high nutritional microalgae biomass with a lower price, biocircular economy approach was integrated; using aquaculture wastewater is one of the main actions to sustain biomass production and minimize environmental impacts.

The potential capacity of integration of microalgae in the aquatic diet is linked to the production of high‐quality compounds by microalgae, including proteins, lipids, carbohydrates, vitamins, minerals, and other nutritional compounds, but one of the most important challenges is to have aquafeeds with high bioavailability for aquatic animals, which requires in advance a preliminary selection of strains with less indigestible compounds. Microalgae application in aquaculture industry could be a beneficial and sustainable aquafeed, with high impact on fish health and quality.

## Author Contributions

**Mohamed Hachimi Alaoui:** writing – original draft, conceptualization, writing – review and editing, methodology, investigation. **Aziz Elmoujtahid:** validation, visualization. **Meriem Bamaarouf:** validation, visualization, supervision. **Hicham El Arroussi**: funding acquisition, supervision, conceptualization, validation, visualization, project administration, writing – review and editing, investigation, methodology.

## Funding

No funding was received for this manuscript.

## Conflicts of Interest

The authors declare no conflicts of interest.

## Supporting Information

Additional supporting information can be found online in the Supporting Information section.

## Supporting information


**Supporting Information** The graphical abstract illustrates the full value chain of microalgae, from cultivation to biomass utilization in aquafeed and nutritional applications, within a sustainable circular bioeconomy framework.

## Data Availability

Data sharing is not applicable to this article as no datasets were generated or analyzed during the current study.
