# Supplementary figures and images for: Exploring the Potential of Microalgae as Feed Ingredients for Sustainable Aquaculture: A Review of Nutritional and Environmental Benefits
Source: Aquac Nutr. 2026 Jan 27;2026:5217142. doi: 10.1155/anu/5217142 (PMC12836876; doi:10.1155/anu/5217142)

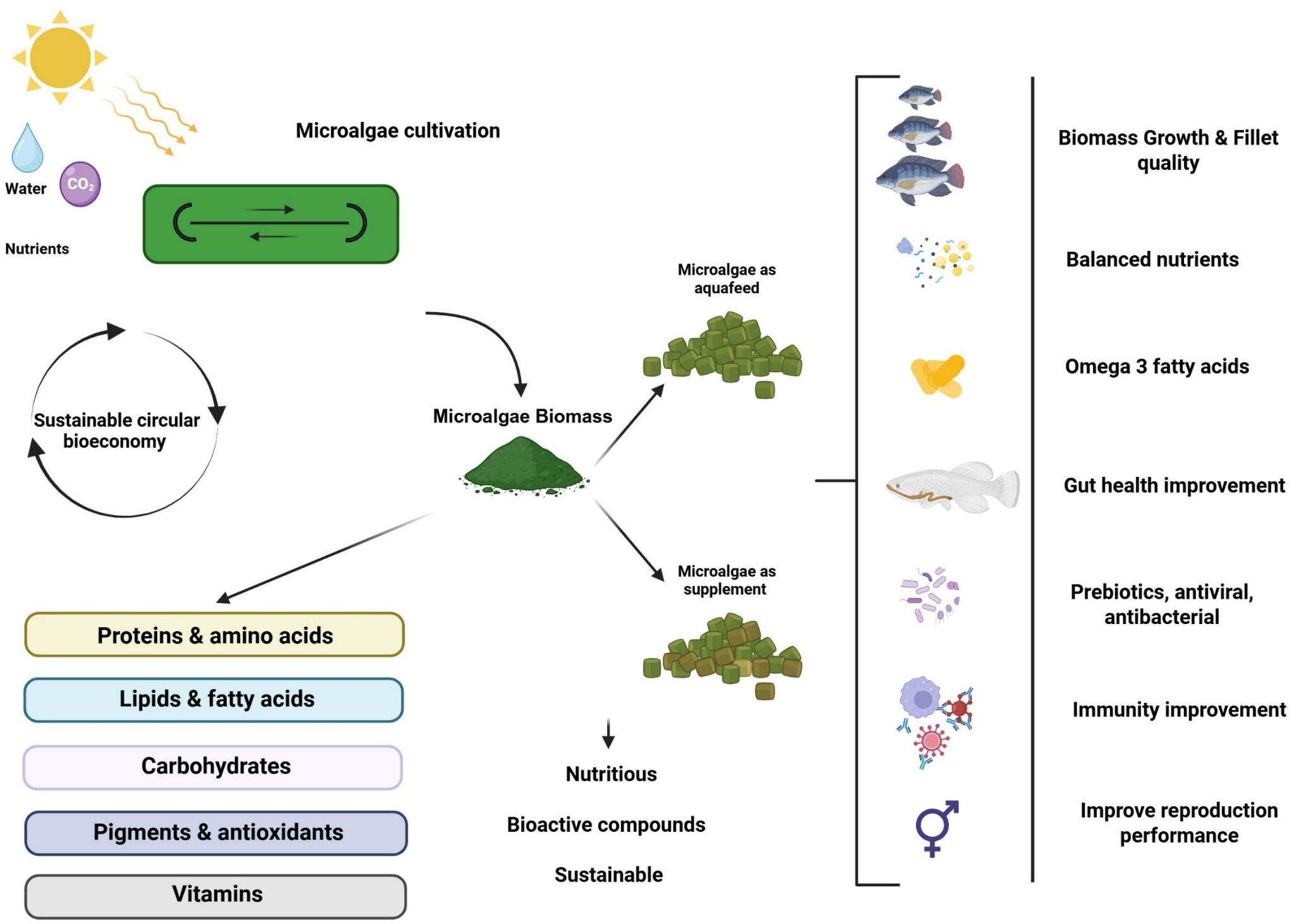

Supplement: Supplementary file 1 — Supporting Information The graphical abstract illustrates the full value chain of microalgae, from cultivation to biomass utilization in aquafeed and nutritional applications, within a sustainable circular bioeconomy framework. [file ANU-2026-5217142-s001.pdf]
